# Comparative analysis on the anti-inflammatory/immune effect of mesenchymal stem cell therapy for the treatment of pulmonary arterial hypertension

**DOI:** 10.1038/s41598-021-81244-1

**Published:** 2021-01-21

**Authors:** Seyeon Oh, Albert Y. Jang, Sehyun Chae, Seungbum Choi, Jeongsik Moon, Minsu Kim, Edda Spiekerkoetter, Roham T. Zamanian, Phillip C. Yang, Daehee Hwang, Kyunghee Byun, Wook-Jin Chung

**Affiliations:** 1 Gachon Cardiovascular Research Institute, Gachon University, Incheon, Republic of Korea; 2 Functional Cellular Networks Laboratory, Lee Gil Ya Cancer and Diabetes Institute, Gachon University, Incheon, Korea; 3 Department of Cardiovascular Medicine, Gachon University Gil Medical Center, Incheon, Republic of Korea; 4 Center for Plant Aging Research, Institute for Basic Science, Daegu Gyeongbuk Institute of Science and Technology, Daegu, Republic of Korea; 5 Korea Brain Bank, Korea Brain Research Institute, Daegu, Republic of Korea; 6 Dongguk University Ilsan Hospital Medical Center, Goyang, Republic of Korea; 7 Department of Cardiology, Hallym University Chuncheon Sacred Heart Hospital, Chuncheon, Republic of Korea; 8 Department of Medicine, Vera M. Wall Center for Pulmonary Vascular Disease, Stanford University, Stanford, CA USA; 9 Division of Cardiovascular Medicine, Stanford Cardiovascular Institute, Stanford University School of Medicine, Stanford, CA USA; 10 Department of Biological Sciences, Seoul National University, Seoul, Republic of Korea; 11 Department of Anatomy and Cell Biology, College of Medicine, Gachon University, Incheon, Republic of Korea

**Keywords:** Biotechnology, Cell biology, Molecular biology, Stem cells

## Abstract

Despite the advancement of targeted therapy for pulmonary arterial hypertension (PAH), poor prognosis remains a reality. Mesenchymal stem cells (MSCs) are one of the most clinically feasible alternative treatment options. We compared the treatment effects of adipose tissue (AD)-, bone marrow (BD)-, and umbilical cord blood (UCB)-derived MSCs in the rat monocrotaline-induced pulmonary hypertension (PH) model. The greatest improvement in the right ventricular function was observed in the UCB-MSCs treated group. The UCB-MSCs treated group also exhibited the greatest improvement in terms of the largest decrease in the medial wall thickness, perivascular fibrosis, and vascular cell proliferation, as well as the lowest levels of recruitment of innate and adaptive immune cells and associated inflammatory cytokines. Gene expression profiling of lung tissue confirmed that the UCB-MSCs treated group had the most notably attenuated immune and inflammatory profiles. Network analysis further revealed that the UCB-MSCs group had the greatest therapeutic effect in terms of the normalization of all three classical PAH pathways. The intravenous injection of the UCB-MSCs, compared with those of other MSCs, showed superior therapeutic effects in the PH model for the (1) right ventricular function, (2) vascular remodeling, (3) immune/inflammatory profiles, and (4) classical PAH pathways.

## Introduction

Pulmonary arterial hypertension (PAH) is a debilitating disease with progressive elevation of the pulmonary artery pressure and vascular resistance, which leads to pulmonary vascular remodeling and right ventricular (RV) failure. Currently approved drugs that modulate the classical pathways (the endothelin, nitric oxide (NO), and prostacyclin pathways) of PAH have shown therapeutic effects, although the mean survival time for a newly diagnosed patient is reported to be only 3 to 7 years^1,2^. Stem cell-based therapies have emerged as an alternative treatment option to the conventional therapies in various cardiovascular diseases^3–7^. It has been shown that stem cells have pleotropic effects on angiogenesis, regeneration, and anti-inflammation in animal models^8,9^. Mesenchymal stem cells (MSCs) are one of the most well-characterized stem cells that are clinically applicable due to their trans-species/individual immune tolerance, rapid proliferation, and easy handling^10–14^. MSCs have also been shown to be pluripotent and to have immune modulatory effects^15^. The therapeutic effects of the MSCs derived from adipose tissue (AD-MSC)^16^, bone-marrow (BM-MSC)^17,18^, and umbilical cord blood (UCB-MSC) have shown promising data in animal models^19^, although head-to-head comparisons between these cell types in a pulmonary hypertension (PH) model have not been investigated. Herein, we compared the therapeutic effects of intravenous (IV) AD-, BM- and UCB-MSCs treatments in a monocrotaline induced PH (MCT + Saline) rat model using echocardiography, histology, immunohistochemistry, and gene expression analysis.

## Results

### Characterization of the cultured MSCs

After culturing AD-, BM-, and UCB-MSCs, we first examined the purity of the cultured MSCs by measuring the expression of a human MSC-specific positive marker (*Cd44* or *Cd90*) and a negative marker (*Cd34*)^15,20^ (sFig. 1). All three MSC cell types showed strong peaks for the positive markers and virtually no signal for the negative marker, suggesting that the cultured MSCs were highly pure human MSCs.

### MSC therapy reverses RV pressure overload and dysfunction caused by PAH

**Figure 1:**
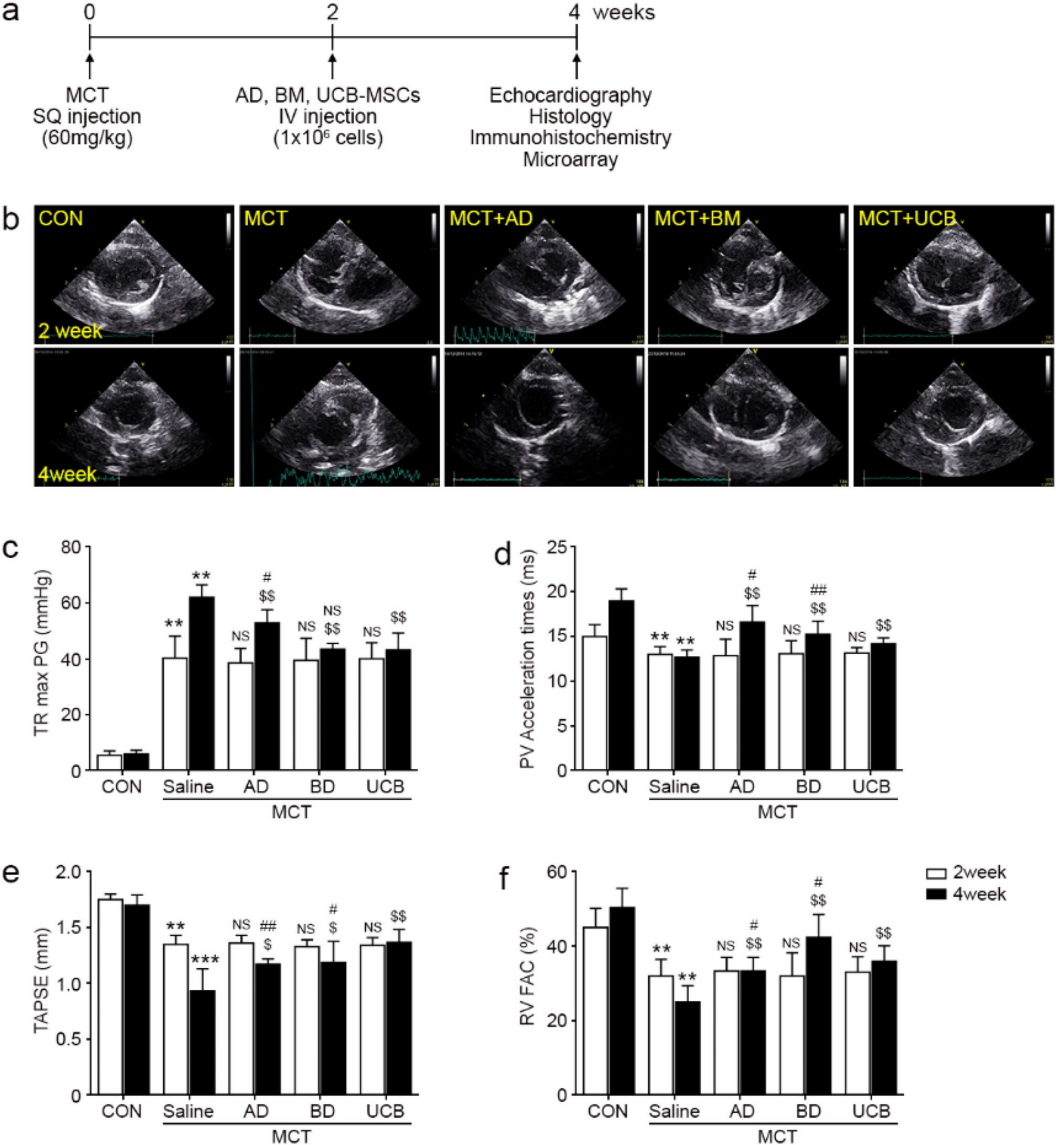
Echocardiographic analysis of MSC-treated lungs at week 2 and 4. (**a**) Schematic illustration of MCT and MSC treatment experiments. MCT was subcutaneously injected into 8-week-old male rats. At 2 weeks post-MCT injection, MSCs (1 × 10^6^ cells) were injected into randomized animals via the tail vein. At 4 weeks post-MCT injection, the echocardiography was performed before the animals were sacrificed for tissue analysis. (**b**) The effects of the MSC administration on right ventricular (RV) pressure and function were evaluated by echocardiography at week 2 and 4 post-MCT injection. Representative images of the short axis view of the echocardiogram showing the right and left ventricle of the rat heart. D-shaped left ventricles (LV) caused by elevated RV pressure were observed in MCT + Saline group, where the MSC injected groups had attenuated RV size and D-shaped compared with the MCT + Saline group. (**c**–**f**) The tricuspid regurgitation maximal pressure gradient (TR max PG), pulmonary velocity acceleration time (PVAT), tricuspid annular plane systolic excursion (TAPSE), and RV fractional area contraction (RV FAC) of each treatment group are shown. Each group was examined in n = 7 mice. Data are shown as the mean ± SD. Statistical significance is marked with 3 symbols. * for the comparison with control; $ for the comparison with the MCT + Saline group; # for the comparison with the MCT + UCB group; *, $, #* P* < 0.05; **, $$, ##* P* < 0.01; ***, $$$, ###* P* < 0.001 from Mann–Whitney test; CON: control ; MCT: monocrotaline; MSC: mesenchymal stem cell; RV: right ventricule; LV: left ventricle; AD: adipose tissue-derived; BM: bone-marrow-derived; UCB: umbilical cord blood-derived; TR max PG: tricuspid regurgitation max pressure gradient; PVAT: pulmonary velocity acceleration time; TAPSE: tricuspid annular plane systolic excursion; RV FAC: right ventricular fractional area contraction.

To evaluate therapeutic effects of each type of MSCs in the MCT model, 1 × 10^6^ cultured MSCs were administered by IV tail injection at 2 weeks post-MCT injection (Fig. 1a). At two weeks post-MSC injection, RV function and pressure overload were assessed by echocardiography (Fig. 1b–f). At week 2, the MCT + Saline group showed an increased tricuspid regurgitation maximal pressure gradient (TR max PG, 61.24 ± 4.31 mmHg) and decreased pulmonary velocity acceleration time (PVAT, 12.65 ± 0.85 ms) were noted compared with the control (CON) group (11.95 ± 3.99 mmHg for TR max PG and 18.99 ± 1.32 ms for PVAT), suggestive of RV pressure overload(Fig. 1c–f). The increased TR max PG was significantly reduced by the administration of the MCT + BM (28.96% reduction, 43.93 ± 2.09 mmHg) and MCT + UCB (35.08% reduction, 39.76 ± 5.08 mmHg) (Fig. 1c). Compared with the BM- and UCB-MSC treatments, the AD-MSC treatment led to a relatively weaker reduction (13.73% reduction, 52.83 ± 4.10 mmHg). The decreased PVAT in the MCT + Saline group was also significantly restored in the MCT + AD (31.38% increase, 16.62 ± 1.85 ms), MCT + BM (20.63% increase, 15.26 ± 1.46 ms) and MCT + UCB (12.41% increase, 14.22 ± 0.61 ms) groups (Fig. 1d). Tricuspid annular plane systolic excursion (TAPSE) and RV fractional area contraction (RV FAC) were decreased in MCT + Saline group (1.71 ± 0. 09 mm for TAPSE and 24.95 ± 4.45% for RV FAC), suggestive of RV dysfunction caused by MCT + Saline (Fig. 1e,f). Decreased TAPSE and RV FAC were also significantly restored in the MCT + AD (28.26% increase for TAPSE and 33.59% increase for RV FAC), MCT + BM (26.09% increase for TAPSE and 69.70% increase for RV FAC) and MCT + UCB (55.43% increase for TAPSE and 44.05% for RV FAC) groups (Fig. 1e,f). These data suggested that the BM- and UCB-MSC treatments had the strongest restorative effect against impaired RV hemodynamics.

### Protective effects of UCB-MSCs on PAH-induced vascular remodeling

**Figure 2:**
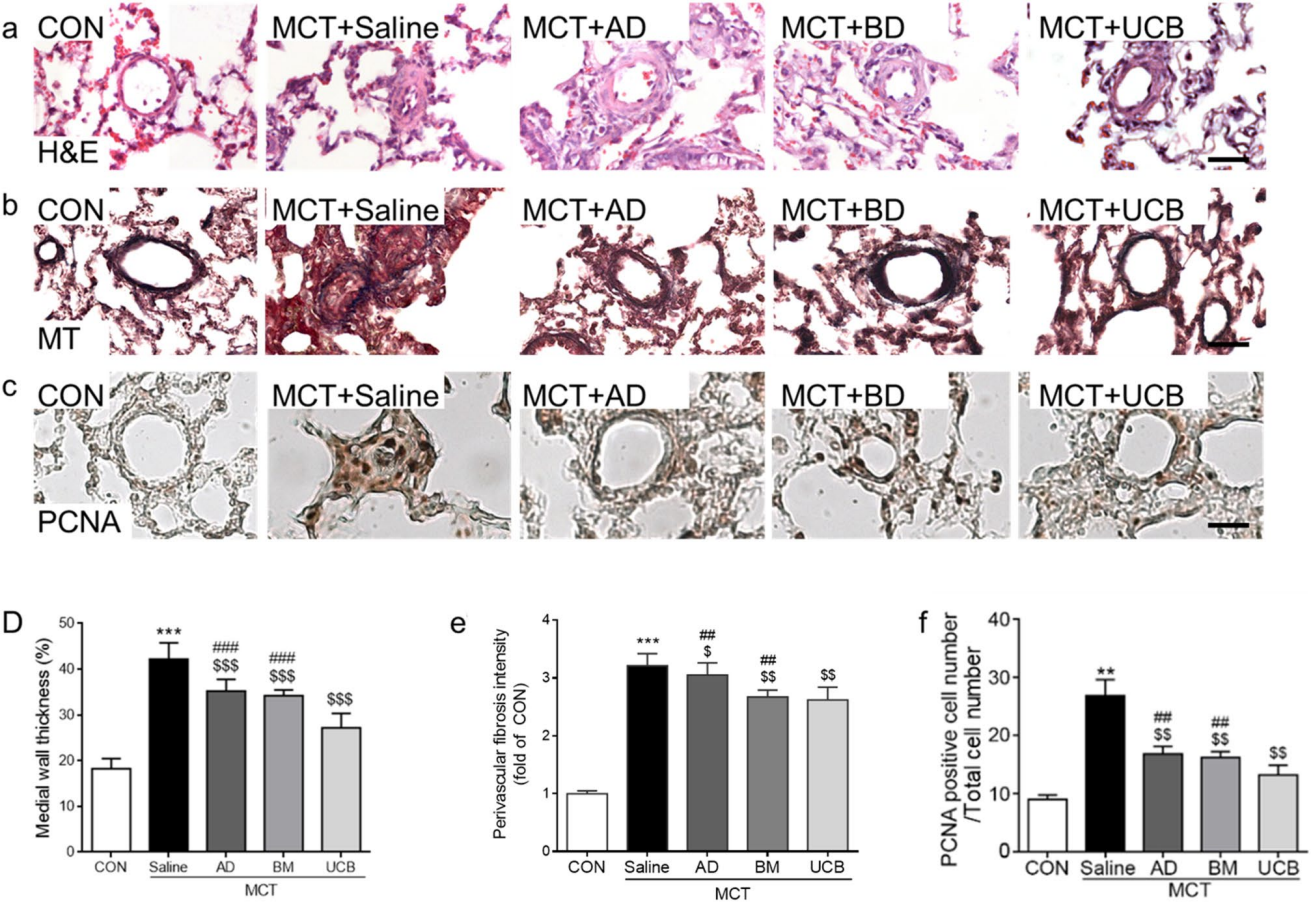
Histological and immunohistochemical analyses of vascular remodeling. (**a**–**c**) Representative images obtained from H&E (**a**), Masson’s Trichrome (**b**), and PCNA staining (**c**), which were used to evaluate the pulmonary arterial media thickness, perivascular fibrosis, and cell proliferation, respectively. The scale bar represents 100 μm. (**d**–**f**) Quantitation of the medial wall thickness (**d**), perivascular fibrosis intensity (**e**), and PCNA-positive cell numbers (**f**). Data are shown as the mean ± SD. Statistical significance is marked with 3 symbols. * for the comparison with the control; $ for the comparison with the MCT + Saline group; # for the comparison with the MCT + UCB group; *, $, #* P* < 0.05; **, $$, ##* P* < 0.01; ***, $$$, ###* P* < 0.001 from Mann–Whitney test; CON: control ; MCT: monocrotaline; AD: adipose tissue-derived; BM: bone-marrow-derived; UCB: umbilical cord blood-derived.

Next, the pulmonary arterial medial wall thickness and perivascular fibrosis in rat lungs were compared between the MCT + Saline and the MCT + MSC-treated groups to assess the therapeutic effects of the MSCs. The MCT treatment significantly increased the medial wall thickness and perivascular fibrosis compared with CON group (Fig. 2a,d). Overall, the treatment of all three MSCs significantly reduced the MCT-induced increase in the media wall thickness for MCT + AD, BM, and UCB groups, respectively (Fig. 2a,d). Intriguingly, the MCT + UCB group showed the most significant the reduction compared with the other two MSC groups. Similarly, the increased perivascular fibrosis in the MCT + Saline group was significantly reduced in the MCT + AD, MCT + BM, and MCT + UCB groups, and the MCT + UCB group had more significant attenuation than the MCT + AD and MCT + BM groups (Fig. 2b,e). Moreover, we next compared the efficacy of the three MSCs in attenuating vascular cell proliferation thorough PCNA, proliferation marker (Fig. 2c,f). The increased percentage of PCNA-positive cells in the MCT + Saline group was significantly reduced in the MCT + AD, MCT + BM, and MCT + UCB groups, and the MCT + UCB group showed the greatest effect (Fig. 2f). These data collectively demonstrated that all three MSC treatments showed protective effects on the medial wall thickening, perivascular fibrosis, and vascular cell proliferation in the MCT + Saline group, and that the MCT + UCB had significantly greater effects than the other two MSC types.

### Effective engraftment of the injected MSCs in lungs

To evaluate the effectiveness of the engraftment of the injected MSCs, we next measured the levels of the human stem cell markers (*CD44, CD90,* CD*29, human nuclear antigen (HNA), and human Arthrobacter luteus (Alu)*) within the MCT-treated lungs harvested at days 1, 3, 5, 7, and 14 post-MSC injection. Their mRNA levels can reflect the amounts of the engrafted MSCs at days post-MSC injection. The mRNA levels of the three markers gradually decreased over time (sFig. 2). Intriguingly, significant levels of the three markers were stably detected up to day 7, suggesting that significant portions of all three MSCs were stably engrafted in the lungs. Among the three MSCs, the MCT + UCB showed the highest mRNA levels of the three markers, especially at days 3 and/or 5 post-MSC injection, suggesting the most effective engraftment of the UCB-MSCs.

### Inhibitory effects of UCB-MSCs on innate and adaptive immunity

**Figure 3:**
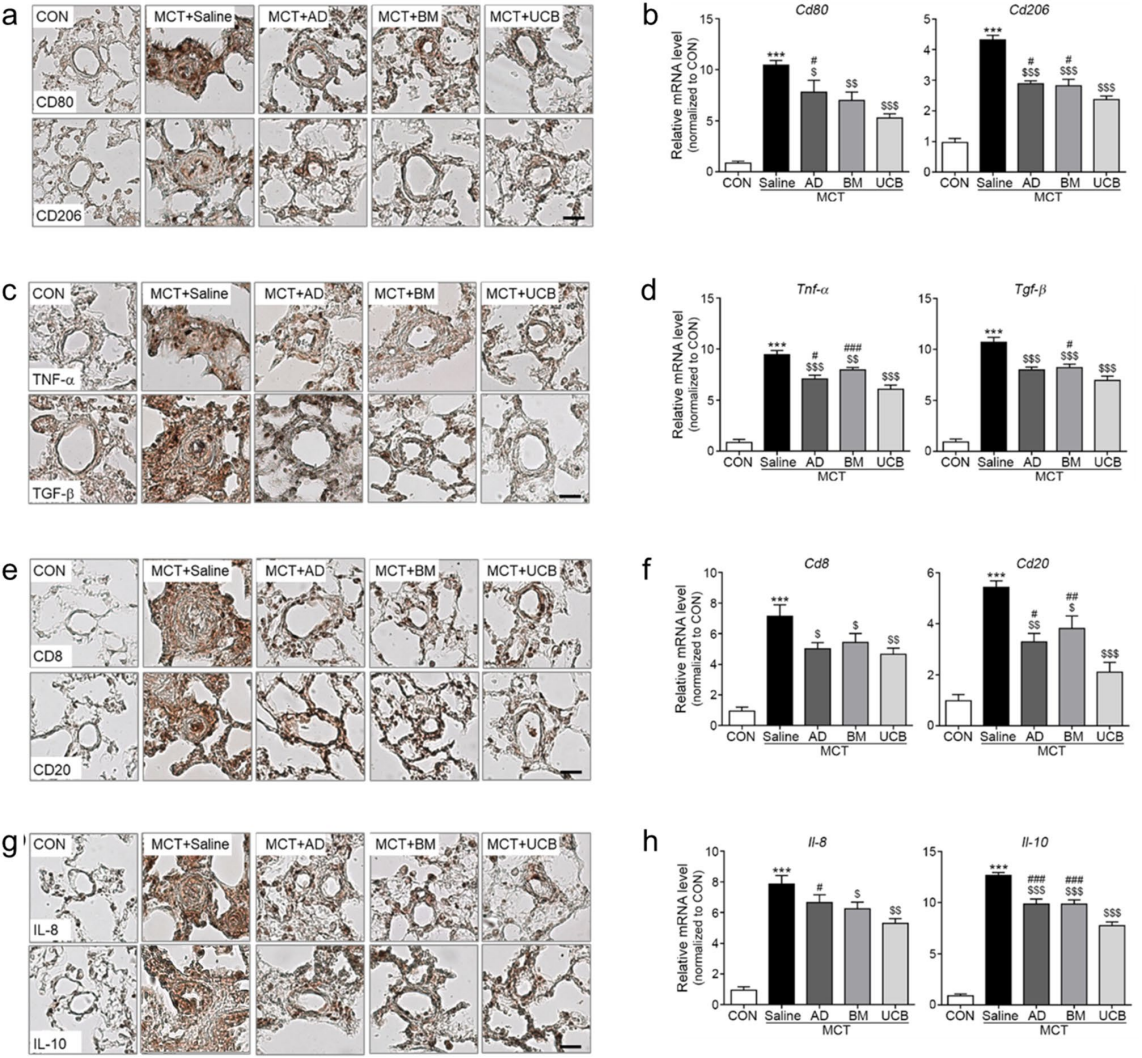
Inhibitory effects of UCB-MSCs on innate/adaptive immunity and inflammation. (**a**,**b**) immunostainings and mRNA expression levels of the markers for M1 macrophages (*Cd80*), and M2 macrophages (*Cd206*). (**c**,**d**) immunostainings and mRNA expression levels of inflammatory cytokines of M1 (*Tnf-α*) and M2 macrophages (*Tgf-β*). (**e**,**f**) immunostsainings and mRNA expression levels of the markers for T cells (*Cd8*) and B cells (*Cd20*). (**g**,**h**) immunostainings and mRNA expression levels of the inflammatory cytokines in T cells (*IL-10*) and B cells (*IL-8*). Data are shown as the mean ± SD. Statistical significance is marked with 3 symbols. * for the comparison with the control; $ for the comparison with the MCT + Saline group; # for the comparison with the MCT + UCB group; *, $, #*P* < 0.05; **, $$, ##*P* < 0.01; ***, $$$, ###*P* < 0.001 by Mann–Whitney test; MCT: monocrotaline; CON: control; AD: adipose tissue-derived; BM: bone-marrow-derived; UCB: umbilical cord blood-derived.

We next compared the level of infiltrated inflammatory cells within the MCT-treated lungs harvested on day 14 post-MSC injection. The immunostainings and mRNA levels of the representative markers *CD80* and *CD206* for the pro-inflammatory macrophage (MΦ) subtype (M1) and pro-fibrotic MΦ subtype (M2), respectively, were significantly increased in the MCT + Saline group. The increase was reduced in all MSC-treated groups, and such reduction was relatively stronger in the MCT + UCB group (Fig. 3a,b). Moreover, we analyzed immunostaining and mRNA levels of the cytokines that represent the activated M1 (*Tnf-α*) and M2 (*Tgf-β*) (Fig. 3c,d). The mRNA levels of these cytokines were increased in the MCT + Saline group. Similarly, the increase was significantly reduced in all the MSC-treated groups, and the reduction was relatively stronger in the MCT + UCB group (Fig. 3c,d). Furthermore, we examined whether the attenuated innate immunity further modulates adaptive immunity by measuring the mRNA levels of *Cd8* and *Cd20*, T and B cell markers, respectively. Immunostainings and mRNA levels of these markers were significantly increased in the MCT + Saline group, although MCT + UCB group had the largest reversing effects (Fig. 3e,f). Similar immunostaining and mRNA expression patterns of *Il-10* and *Il-8* that represent T and B cell activations, respectively, were observed in the MCT + Saline and MSC-treated groups (Fig. 3g,h). Collectively, these data suggest that UCB-MSCs more strongly attenuate innate and adaptive immunity associated with inflammation and fibrosis in the MCT + Saline model.

### Modulation of PAH-related processes by the MSCs

**Figure 4:**
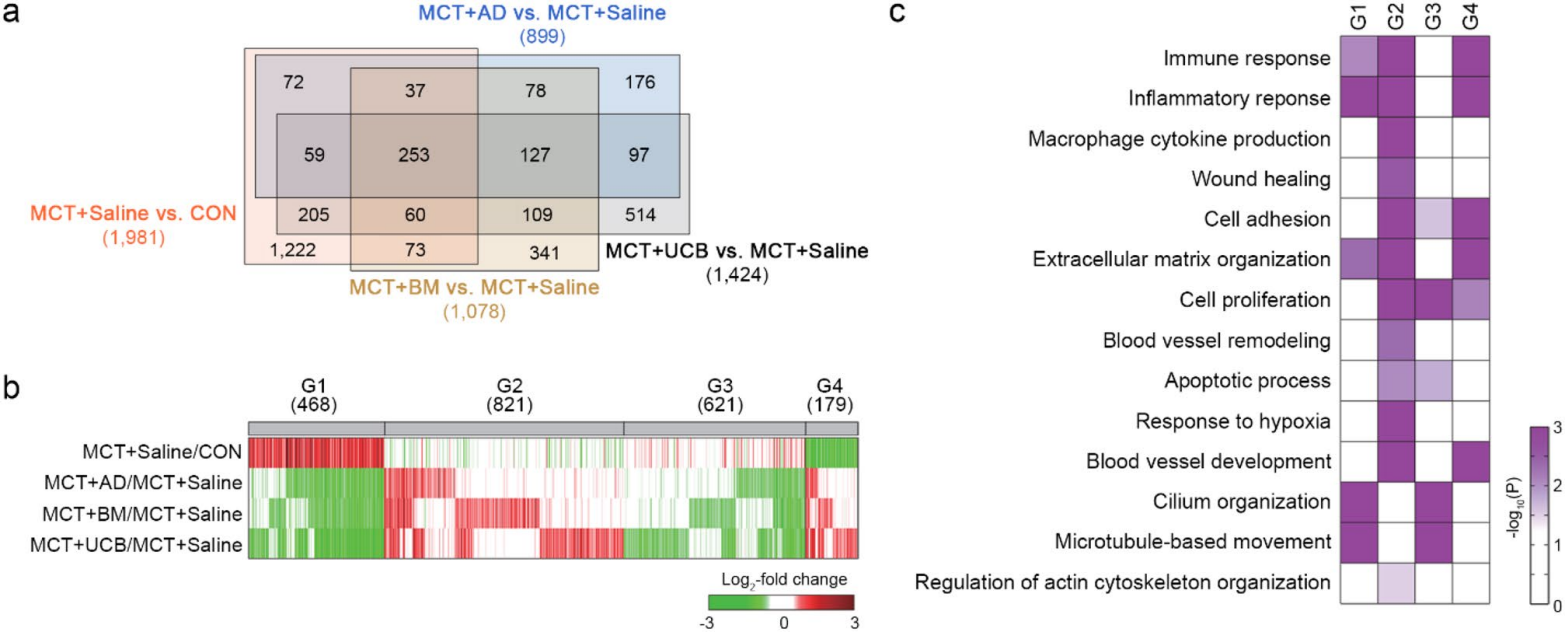
Cellular processes affected by AD-, BD-, and UCB-MSCs in the MCT model. (**a**) Venn diagram showing the relationships among the DEGs from the four indicated comparisons. Numbers in parentheses represent the numbers of DEGs identified from the comparisons. (**b**) Heat map showing the differential expression of the genes in G1–4. The colors indicate up-regulation (red) and down-regulation (green) in MCT, and AD-, BD-, and UCB-MSCs, compared to the control conditions in the corresponding comparisons. The color bar represents the gradient of the log_2_-fold-changes in the comparisons. Numbers in parentheses represent the numbers of DEGs in the indicated groups. (**c**) GOBPs represented by the genes in G1–4. The color bar represents the gradient of − log_10_(p), where p is the significance of the individual GOBPs that are enriched by the DEGs in G1–4. The heat map was generated using MATLAB (imagesc.m in R2019a; www.mathworks.com/). DEG: differentially expressed genes; GOBP: gene ontology biological processes; MCT: monocrotaline; CON: control; AD: adipose tissue-derived; BM: bone-marrow-derived; UCB: umbilical cord blood-derived.

We next conducted gene expression profiling of the MSC-treated lung tissues to understand the molecular nature underlying the MSC-induced attenuation of PAH phenotypes in the MCT + Saline model. To identify the genes affected by MCT and the MSCs, we performed the following four comparisons: (1) MCT versus saline control (MCT + Saline/CON), (2) AD-MSC versus MCT (MCT + AD/MCT + Saline), (3) BM-MSC versus MCT (MCT + BM /MCT + Saline), and (4) UCB-MSC versus MCT (MCT + UCB/MCT + Saline). A total of 3423 differentially expressed genes (DEGs) with an FDR ≤ 0.05 were identified from these comparisons, including 1981, 899, 1078, and 1424 DEGs for MCT + Saline/CON, MCT + AD/MCT + Saline, MCT + BM/MCT + Saline, and MCT + UCB/MCT + Saline, respectively (Fig. 4a). The DEGs were subsequently categorized into 8 groups (G1–8) based on their up- or down-regulation patterns by MCT + Saline and/or the MSCs (sFig. 3). Among G1–8, we focused on the four groups (G1–4) whose expression levels were differently changed by MCT + Saline and the MSCs, which can explain the effects of the MSCs in the MCT + Saline model (Fig. 4b). The largest number of DEGs were identified from MCT + UCB/MCT + Saline (Fig. 4a), and G1–4 included a larger number of DEGs from MCT + UCB/MCT + Saline compared with those from MCT + AD/MCT + Saline and MCT + BM/MCT + Saline, suggesting that UCB-MSCs most strongly affected gene expression in the MCT + Saline model (Fig. 4b). Next, we identified cellular processes associated with G1–4 by performing enrichment analysis of GOBPs using the DAVID software^21^ (Fig. 4c). G1–4 were strongly associated with PAH-related cellular processes, such as (1) inflammation (inflammatory response, Mɸ cytokine production, and wound healing); (2) vascular remodeling (cell adhesion, extracellular matrix organization, cell proliferation, and blood vessel remodeling); (3) angiogenesis (response to hypoxia and blood vessel development); (4) apoptosis (apoptotic signaling pathway); and/or (5) cytoskeleton organization (cilium organization, microtubule-based movement, and regulation of actin cytoskeleton organization. Collectively, these data suggest that UCB-MSCs most greatly modulate PAH-related process including inflammation and vascular remodeling (Fig. 4c).

### Predominant effects of UCB-MSCs on inflammatory response

**Figure 5:**
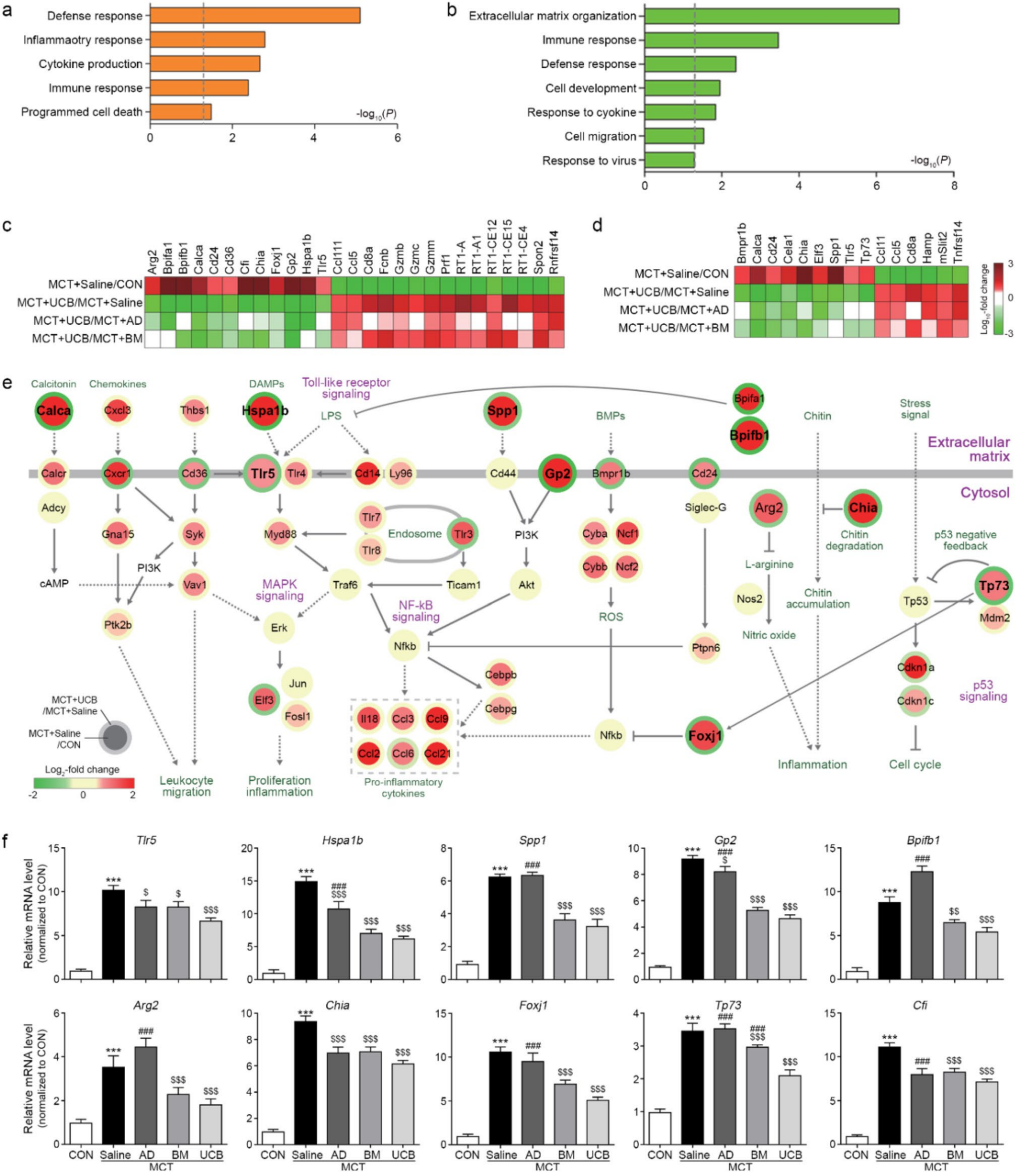
Inhibitory effects of UCB-MSC administration on the immune and inflammatory responses. (**a**,**b**) GOBPs represented by the upregulated (**a**) and downregulated (**b**) genes predominantly by UCB-MSCs. Enrichment significance (p) for each GOBP is displayed in − log_10_(p). (**c**,**d**) Heat maps showing the differential expression of the genes involved in the inflammatory (**c**) and immune responses (**d**). The color bar represents the gradient of the log_2_-fold-changes in the comparisons. The heat map was generated using MATLAB (imagesc.m in R2019a; www.mathworks.com/). (**e**) A network model describing interactions among the predominantly upregulated genes by UCB-MSCs. Node center and boundary colors represent upregulation (red) and downregulation (green) by MCT and UCB-MSCs, respectively. Solid and dotted arrows (or inhibition symbols) denote direct and indirect activations (or inhibition), respectively. The color bar represents the gradient of log_2_-fold-changes in the corresponding comparison (MCT + Saline/CON or MCT + UCB/MCT + Saline). The network model was generated using Cytoscape software (v.3.3.0; www.cytoscape.org/). (**f**) Confirmation of the predominant upregulation of the indicated three representative genes involved in the inflammatory and immune responses by qRT-PCR. The expression levels were normalized with respect to those in the control group. The normalized data are expressed as the mean ± SD. Statistical significance is marked with 3 symbols. * for the comparison with the control; $ for the comparison with the MCT + Saline group; # for the comparison with the MCT + UCB group; *, $, #*P* < 0.05; **, $$, ##*P* < 0.01; ***, $$$, ###*P* < 0.001 by Mann–Whitney test; GOBP: gene ontology biological processes; MCT: monocrotaline; CON: control; AD: adipose tissue-derived; BM: bone-marrow-derived; UCB: umbilical cord blood-derived.

As UCB-MSCs showed more inhibitory effects on PAH phenotypes compared with AD- and BM-MSCs, we next identified the genes showing up- or down-regulation in the MCT + UCB group compared with the MCT + AD and BM groups. To this end, among the 468 up-regulated genes by MCT + Saline in G1 and 179 down-regulated genes in G4 (Fig. 4b), we first identified 380 (G1) and 135 genes (G4) that showed the reversion of the MCT-induced expression changes by UCB-MSCs (sFig. 4a). Of these genes, we further selected 108 (G1) and 88 genes (G4) that showed the stronger reversion of the MCT-induced expression changes by UCB-MSCs than by AD- and BM-MSCs through the comparisons of UCB-MSC versus AD-MSC (MCT + UCB/MCT + AD) and UCB-MSC versus BM-MSC (MCT + UCB/MCT + BM) (sFig. 4a). The enrichment analysis of the GOBPs revealed that the 108 up-regulated genes were mainly involved in cellular processes related to inflammatory response (defense, inflammatory, and immune responses, and cytokine production) (Fig. 5a). The 88 down-regulated genes were mainly involved in the processes related to immune response (defense response, and responses to cytokine and virus), cell development and migration, and extracellular matrix organization (Fig. 5b). Together with the findings in Fig. 3, these data suggest that the inflammation and immune response-related processes may play critical roles in the inhibitory effects of UCB-MSCs on PAH phenotypes.

Next, we thus selected 27 genes related to inflammation (Fig. 5c) and 15 genes related to immune and defense responses (Fig. 5d) and found that they included both upregulated and downregulated genes by MSCs. To sort out cellular processes upregulated and downregulated predominantly by UCB-MSCs in the MCT-treated condition, we built network models describing interactions among these upregulated (Fig. 5e) or downregulated (sFig. 4b) genes involved in the inflammatory and immune responses. The network model for the upregulated genes (Fig. 5e) showed that UCB-MSCs predominantly downregulated pro-inflammatory pathways activated by MCT, including damage-associated molecular patterns, such as heat shock protein A member 1b (*Hspa1b*), its receptor [toll-like receptor 5 (*Tlr5*)), and other modulators and their cofactors (*Arg2*, *Chia, Spp1*, and *Gp2*), as well as resolution of inflammation (*Bpifa1/b*). The UCB-MSCs also negatively regulated of the MCT-induced T and B cell activation (*Tp73*, and *Foxj1*). These data suggest that UCB-MSCs, compared with AD- and BM-MSCs, more strongly decrease the inflammatory responses induced by MCT + Saline in general, including early inflammation and late resolution of inflammation, as well as activation of adaptive immune responses (T and B cell activations). On the other hand, the network model for the downregulated genes (sFig. 4b) showed that UCB-MSCs predominantly upregulated the pathways induced by pathogen infections, which were downregulated by MCT, including complement cascade (*Fcnb* and *C4bpa*), NK cell activation (*Klrb1a/c*, *Spon2*, and *Slit2*), cytotoxic T cell-mediated cell killing pathways-MHC1 class 1 presentation (*RT1-A/A1*, *RT1-CE2/3/4/12/14/15/16*, *RT1-S2/3*, and *Tap1/2*), and killing of target cells (*Gzma/b/c/k/m*, and *Prf1*). These data suggest that the injected MSCs are killed as foreign cells by these pathways, and the predominant activation of these pathways in the MCT + UCB group reflects a prolonged survival of UCB-MSCs, compared with AD- and BM-MSCs, consistent with the findings illustrated in sFig. 2.

Finally, we confirmed stronger upregulation and downregulation of the representative genes involved in inflammatory and immune responses (*Arg2*, *Hspa1b*, *Spp1*, *Gp2*, *Bpifb1*, *Chia*, *Tp73*, *Foxj1*, and *Cfi*; Fig. 5F) and killing of target cells (*Fcnb*, *Spon2*, *RT1-A*, *RT1-CE4*, and *RT1-CE12*; sFig. 4c) in the MCT + UCB group, compared with in the MCT + AD and BM groups. Taken together, all these data suggest that UCB-MSCs, compared with AD- and BM-MSCs, more strongly suppress the progression of inflammation induced by MCT and thereby the resolution of inflammation as well.

### Inhibitory effects of UCB-MSCs on PAH progression through the PAH and inflammation-related pathways

**Figure 6:**
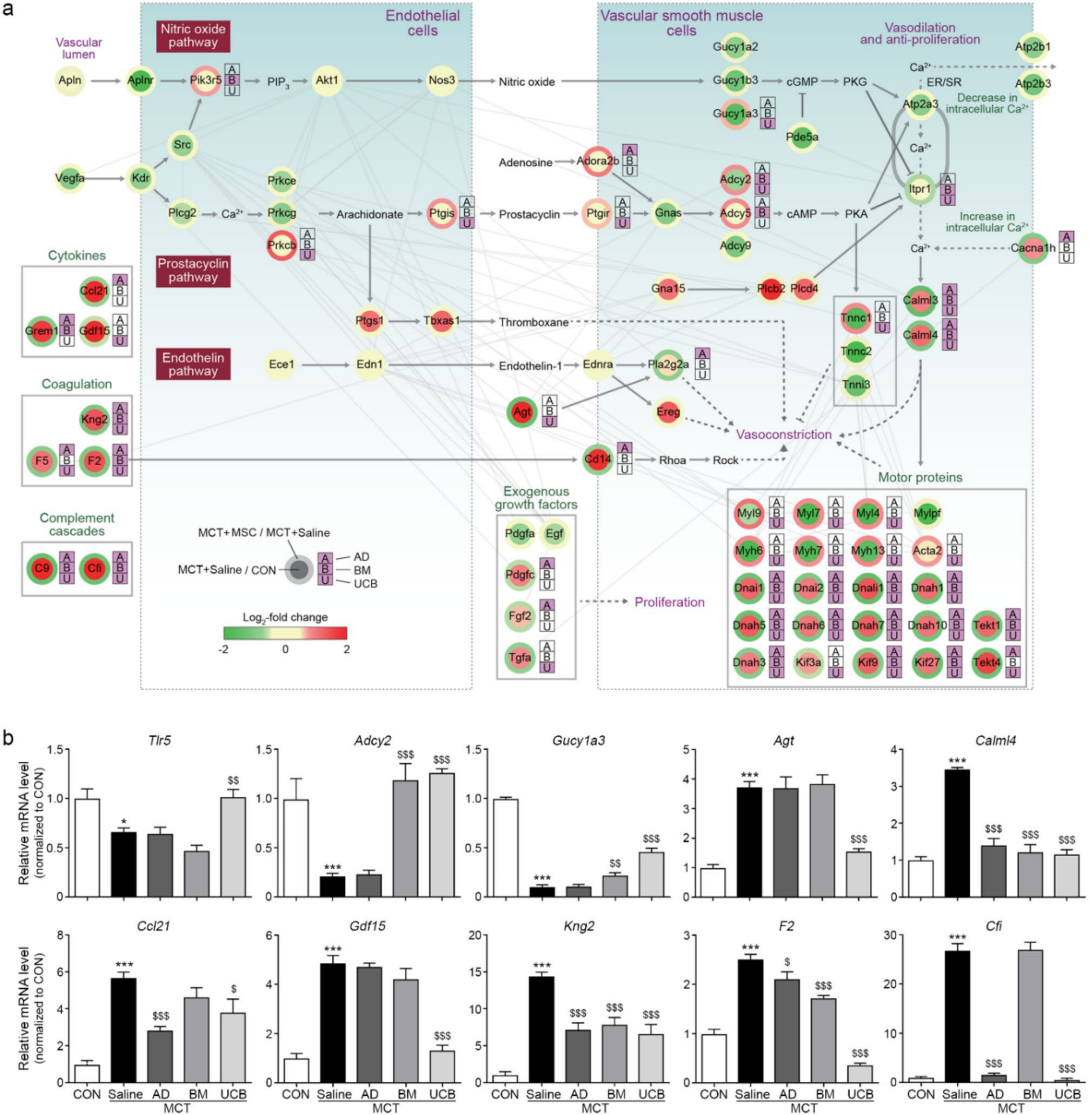
Inhibitory effects of UCB-MSCs on PAH progression. (**a**) Network model describing modulation of PAH and inflammation-related signaling pathways by the MSCs. Node center and boundary colors represent upregulation (red) and downregulation (green) by MCT and UCB-MSCs, respectively. The color bar represents the gradient of log_2_-fold-changes in the corresponding comparison (MCT + Saline/CON or MCT + UCB/MCT + Saline). The stacked bar shows which MSC significantly affected the corresponding genes: for example, ‘U’ in purple indicates the corresponding gene was identified as a DEG in the comparison of MCT + UCB versus MCT + Saline. The color bar denotes the gradient of the log_2_-fold-changes in the four comparisons. Edges represent the protein–protein interactions (gray) between the genes in the network model, which were collected from the five interactome databases. Solid and dotted arrows (or inhibition symbols) denote direct and indirect activations (or inhibition), respectively. The network model was generated using Cytoscape software (v.3.3.0; www.cytoscape.org/). (**b**) Confirmation of the differential expression of the indicated representative genes involved in the PAH and inflammation-related pathways. The expression levels were normalized with respect to those in the CON group. The normalized data are expressed as the mean ± SD.* for the comparison with the control; $ for the comparison with the MCT + Saline group; # for the comparison with the MCT + UCB group; *, $, #*P* < 0.05; **, $$, ##*P* < 0.01; ***, $$$, ###*P* < 0.001 by Mann–Whitney test; DEG = differentially expressed genes; MCT = monocrotaline; CON = control; AD = adipose tissue-derived; BM = bone-marrow-derived; UCB = umbilical cord blood-derived.

To understand the effects of the MSCs on the classical PAH pathways (prostacyclin, endothelin, and NO pathways), we reconstructed a network model describing how the MSCs modulated the MCT-induced expression changes of the components in the PAH pathways. The network model showed down-regulation of prostacyclin and NO pathways (*Gucy1a2*/*a3*/*b3* in NO pathway and *Gnas* and *Adcy2*/*9* in prostacyclin pathway) by MCT, which promote vasodilation (Fig. 6a, upper part). In contrast, the network model showed up-regulation of endothelin pathway (*Ereg* and *Agt*) by MCT, which promotes vasoconstriction, consistent with the previous findings (Fig. 6a, middle part)^22^. Intriguingly, these alterations in the three pathways were restored by the MSCs (*Gucy1a3* in NO pathway; *Prkcb*, *Ptgir/s*, *Adora2b*, and *Adcy2/5* in prostacyclin pathway; and *Agt* and *Pla2g2a* in endothelin pathway). Moreover, the network model showed the effects of the MSCs on Ca^2+^ signaling pathway that closely interacts with the above PAH pathways (Fig. 6a, right part). Ca^2+^ transporters were altered (down-regulation of *Atp2a3*/*2b1*/*2b3* and up-regulation of *Cacna1h*) by MCT to increase the cytosolic Ca^2+^ concentration and activate Ca^2+^ signaling (*Calml3*/*4*) and motor protein complexes (*Dnas* and *Kifs*), which promote vasoconstriction. These alterations were also restored by the MSCs (*Cacna1h* and *Calml3*/*4*). Furthermore, the network model showed up-regulation of inflammation-related pathways, cytokine (*Ccl21*, *Grem1*, and *Gdf15*) and coagulation factor (*Kng2* and *F2/5*) pathways by MCT, which can promote vasoconstriction (Fig. 6a, left). These alterations were also restored by the MSCs. These data suggest that the MSCs can contribute to the restoration of the MCT-induced changes in the PAH and inflammation pathways. However, the comparison of the numbers of the components in the network model reversed by the individual MSCs revealed that UCB-MSCs (38 components) had the largest number of the components affected by MCT compared with AD- (28 components) and BM-MSCs (23 components). Next, we validated these MSC-induced recovery patterns caused by MCT for the following representative components in the PAH or inflammation pathways by qRT-PCR analysis: (1) *Ptgis* and *Adcy2* in the prostacyclin pathway; (2) *Gucy1a3* in the NO pathway; (3) *Agt* in the endothelin pathway; (4) *Calml4* in the Ca^2+^ signaling pathway; and (5) *Ccl21*, *Gdf15*, *Kng2*, *F2*, and *F5* in inflammation pathways (Fig. 6b). Overall, all these data suggest that MSCs reverse the MCT-induced changes in the PAH and inflammation pathways. The UCB-MSCs, among the MSCs, can serve as the most potent MSC with such antagonizing effects.

## Discussion

Despite the recent advent of the use of stem cell paracrine factors, such as exosomes and small molecules, as a treatment option in cardiovascular medicine^23,24^, MSC cell therapy remains a potent and valuable biological treatment option. Although cell-free exosome treatment has been shown to replicate the effects of stem cell therapy to a certain extent, the heterogeneity of the exosomes, low reproducibility, and short half-life bring into question whether the treatment effects could be consistently sustained^25–27^. Doubts have also been raised about whether small molecules derived from stem cells, such as microRNAs, could outperform the pleotropic effects of stem cells. Embryonic stem cells can give rise to virtually all cell lineages, although they are predisposed to teratoma formation and are difficult to obtain without encountering ethical issues^28,29^. The discovery of induced pluripotent stem cells has resolved such ethical issues^29^, but the genetic instability observed under in vitro culture conditions, expensive media costs, propensity for teratoma formation, and relatively long period required for cell reprogramming have been obstacles for their immediate clinical application^30–32^. MSCs are free from ethical issues^29^, easy to obtain from human tissues^10,13^, immune tolerant between species or individuals^11,12,14^, stable under various types and lengths of in vitro culture, and able to be rapidly expanded in an economic fashion^10,13^, which makes them an attractive treatment option.

The most widely investigated MSCs are the AD-, BM-, and UCB-MSCs. All three MSC types have shown promising treatment effects in animal PH models, although a comparison between the three has not been made to date^16,19,33,34^. We therefore systematically performed a comparative analyses of PAH phenotypes (inflammatory profiles, histological features of vascular remodeling, and echocardiographic parameters of the RV), engraftment features, genome-wide mRNA expression profiles, and the associations with the classical PAH cellular pathways. The MSCs significantly restored the PAH phenotypes (Fig. 1–3), modulated PAH-related cellular processes (Fig. 4), and interacted with the classical PAH pathways (NO, prostacyclin, and endothelin) and inflammation-related pathways (Fig. 6). Interestingly, the tissue transcriptome revealed that a substantial portion of the genes that showed significantly different expression compared with the MCT group were genes involved in the immune response and inflammation (Fig. 4). The attenuation of the recruitment of total alveolar macrophages and the M1 and M2 subsets (Fig. 3a) are associated with the decrease of both pro- and anti-inflammatory responses (Fig. 3b)^35^. The subsequent decrease of adaptive immunity was substantiated by the diminished recruitment of T and B cells (Fig. 3c) and the levels of cytokines (Fig. 3d). These features support the immunomodulatory role of MSCs in PAH.

Of the 3 types of MSCs, the UCB-MSCs showed the most promising results for the RV pressure overload and function (Fig. 1), histological properties (Fig. 2), engraftment (sFig. 1), immune/inflammation modulation (Figs. 3, 5), and classical PAH pathways (Fig. 6). The UCB-MSCs had the most potent effects in attenuating the inflammatory and innate immune responses, and in subsequently decreasing the expression of T and B cell markers, which may be the result of a reduction in the recruitment of lymphocytes due to the decreased activation of MΦ. Moreover, according to the network model, the UCB-MSCs had the most significant effect in restoring the PAH and inflammation-related pathways (Fig. 6). These controls of the immune responses by the UCB-MSCs may have led to the better engraftment profiles in MCT + UCB group, for which the engrafted cell survival was the highest. Interestingly, the potent immunomodulatory effects of the UCB-MSCs could be translated to improved RV pressure overload and function as shown in Fig. 1c–f.

The exact mechanisms behind the anti-inflammatory effects of MSCs and improved heart function are not clearly understood. The reduced pro-inflammatory factors predominantly by UCB-MSCs may act as a potential link of the inflammation to the alleviation of the RV pressure and volume overload. Gene expression profiling and network analysis showed that the modulation of pro-inflammatory factors (*Arg2*, *Hspa1b*, *Tlr5*, *Spp1*, and *Gp2*) genes may help delineate the attenuated immune response and anti-inflammatory phenotypes that we observed in the MCT + UCB group. In particular, *Tlr5* has been shown to be expressed in the intestinal epithelia and in endothelial cells that recognize bacterial flagellin and further to mediate the innate immune responses^36^. Recently, the involvement of *Tlr5* in inflammatory diseases such as rheumatoid arthritis was also discovered^37,38^. Although the role of *Tlr5* in PAH has not been elucidated, our data show that the expression level of *Tlr5* is increased in the MCT + Saline group, and such increase is normalized by the MSC treatment, with the most prominent effect observed in the MCT + UCB group. This may suggest that the *Tlr5*-mediated innate immune response may play a role in the pathogenesis of PAH and that the down-regulation of *Tlr5* may contribute to an attenuation of the development of PAH. It has been also suggested that *Tlr5* may be a *Tnf-α* responsive gene^37^, indicating that the attenuated *Tlr5* levels may reflect the low *Tnf-α* levels in the MCT + UCB group. Future studies are warranted to validate the relationship between *Tlr5* and *Tnf-α.*

G2 showing no change by MCT, but upregulation by MSCs in the MCT-treated condition was associated with PAH-related processes (immune and inflammatory responses, cytokine production, cell proliferation, blood vessel development, and response to hypoxia), suggesting that MSCs induce MCT-independent upregulation of these processes. This observation seems inconsistent with the results presented in Fig. 3. Unlike the MCT-independent upregulation of PAH-related processes in G2, however, the results in Fig. 3 represent restoration of the MCT-dependent upregulation of PAH-related processes by MSCs. These PAH-related processes were represented by the genes in G1 (upregulated by MCT and downregulated by MSCs), G2, and/or G4 (downregulated by MCT and upregulated by MSCs). Network analysis showed that G1 was mainly associated with inflammatory responses induced by MCT, including early inflammation, late resolution of inflammation, and activation of adaptive immune responses, whereas G4 was associated with cytotoxic immune response related to killing of the injected MSCs. Interestingly, G2 was predominantly associated with killing of the injected MSCs (e.g., macrophage activation leading to activation of NK and cytotoxic T cells, as well as neutrophil and B cells; sFig. 4), which is closely linked to the network model for G4 (sFig. 4b). Moreover, the genes involved in blood vessel development in G2 were not closely associated with a central VEGF signaling in angiogenesis, suggesting that they are the indirect angiogenic factors related to immune responses. Also, the genes involved in blood vessel development (45.1%, 23 of 51 genes) and cell proliferation (42.0%, 55 of 131 genes) in G2 overlapped with those involved in immune and inflammatory responses, suggesting that the above conclusion (i.e., killing of the injected MSCs) for the immune and inflammatory responses may apply to these processes.

Previous studies have shown that MSCs derived from human embryonic stem cells (hESCs)^39^ or induced-pluripotent stem cells (iPSCs)^40,41^ show potent therapeutic effect. The MSCs, however, have advantages over its other two counterparts for several reasons, as the clinical availability of hESCs and iPSCs can be a major setback. The hESCs can give rise to virtually all cell lineages, although they predispose to teratoma formation and are difficult to obtain without encountering ethical issues^28,29^. The discovery of the iPSCs has resolved such ethical issues^29^, but genetic instability under in vitro culture conditions, expensive media costs, propensity for teratoma formation, and a relatively long period required for cell reprogramming have been obstacles for immediate clinical application^30–32^. MSCs are free from ethical issues^29^, easy to obtain from human tissues^10,13^, immune tolerant over species or individuals^11,12,14^, are stable under various and prolonged in vitro cultures conditions, and can be rapidly expanded economically^10,13^, which makes it an attractive treatment option.

The data from our study were obtained from the MCT- + Saline PH model, which may not entirely reflect the true patholobiology of human PAH. The lack of validation of our findings in other robust models, such as the hypoxic model or the Sugen-hypoxia model, may undermine the validity of our study. Further studies extending our findings in the hypoxic and sugen-hypoxia model are warranted. Additionally, we were not able to confirm our echocardiographic estimates of RV systolic pressure (RVsP) through direct right heart catheterization (RHC). Despite its conveniences in estimating RVsP or mean pulmonary artery pressure, the diagnostic accuracy of echocardiography has been a concerning issue^42^, leading to guidelines recommending right heart catheterization (RHC) as a gold standard for confirming the diagnosis of pulmonary hypertension^43^. Experimental PH models have also confirmed their findings using RHC^44^. Accordingly, we acknowledge that the lack of validation by direct pressure measurement is a major limitation of our study. Our data in this sense should be reviewed with caution.

In this study, we focused on the comparison of the effects of three different representative MSCs on PAH phenotypes and the restoration of the MSC-perturbed gene expression profiles, and found that UCB-MSCs, among the MSCs tested, had the most potent protective effects on PAH phenotypes, as well as the association of the UCB-MSC-induced gene expression changes (PAH and/or inflammation-related pathways) with the improved PAH phenotypes. Although it can be considered valuable as an initial comparative study, further mechanistic studies are warranted to elucidate the functional link of the attenuated inflammation to the improved PAH phenotypes.

In conclusion, the present comparative, systemic analysis of AD-, BM- and UCB-MSC cell therapy in the rat MCT model demonstrated that although all 3 MSC types had therapeutic benefits, the UCB-MSC treatment showed the most promising features in terms of the (1) RV function, (2) histological features, (3) cell engraftment, (4) classical PAH pathways, and (5) immune/inflammatory responses. Due to the lack of direct hemodynamic pressure measurement, however, the current data needs to be interpreted with caution.

## Methods

This study was identified the institutional and/or licensing committee approving the experiments, including any relevant details and confirmed that all experiments were performed in accordance with relevant guidelines (ARRIVE guidelines) and regulations.

### Animal model of PH

Eight-week-old Sprague Dawley male rats (250–300 g) used in this study were in a pathogen-free and climate-controlled facility with a 12-h light/dark cycle and were fed ad libitum throughout the experiment. The study was approved by the Center of Animal Care and Use of Gachon University (AAALAC; approval number LCDI-2013-0015). The PAH rat model was induced by a single subcutaneous injection (60 mg/kg) of MCT (cat. C2401; Sigma-Aldrich, USA)^39,40^. The control (CON) and MCT + Saline group (n = 7) was injected with saline. AD and UCB-derived MSCs were purchased from Medipost (Seongnam, Korea) and BM-derived MSCs were purchased from Cefobio (Seoul, Korea). At two weeks post-MCT injection, 1.0 × 10^6^ AD- (n = 7), BM- (n = 7), and UCB- (n = 7) MSCs were administered by an IV tail vein injection. Echocardiography, tissue harvesting for immunohistochemistry, histology and microarray were performed at the fourth week post-MCT injection, as shown in Fig. 1a. The survival of engrafted MSCs were assessed by measuring human MSC markers, such as *Cd44, Cd90, Cd29, HNA*, and *Alu* at day 1, 3, 5, 7, and 14 after MSC injection (sFig. 2).

### Echocardiography

After rats were anesthetized with isoflurane, transthoracic echocardiography was performed using a 12 MHz probe (GE Healthcare) and a Vivid Q (GE Healthcare, Israel). The assessed transthoracic echocardiographic parameters included the maximum TR max PG, TAPSE, PVAT, and RV FAC^45^. TR max PG and TAPSE was measured in the apical 4 chamber view, whereas the PVAT and RV FAC was evaluated in the short-axis view^46^. Echocardiographic parameters were measured following the recommendations of the American Society of Echocardiography. The pulmonary artery systolic pressure was calculated as 4V^2^ + right atrial pressure, where V was the maximal velocity of the tricuspid regurgitated jet^39^. The TR max PG and PVAT were measured to estimate RV pressure overload, whereas TAPSE and RV FAC were were examined as indicators of RV functional. The sample sizes of each treatment arm were n = 7 for CON, n = 7 for the MCT + Saline, n = 7 for MCT + AD, n = 7 for MCT + BM, and n = 7 for MCT + UCB.

### Paraffin-embedded tissue section processing

The collected lung tissues are washed with cold PBS and stored in 4% paraformaldehyde at 4 °C for 12 h. The fixed tissue is washed for 12 h for embedding and a paraffin block was processed using a tissue processor. The paraffin blocks were cut to 7 μm using a Microtome and dried at 40 °C for 24 h. The paraffin blocks were subjected to xylene and four concentrations of ethanol (100%, 95%, 80%, and 70%) for dyeing preparation.

### Hematoxylin eosin staining

Paraffin-embedded lung tissues were sectioned with a thickness of 7 μm and used for histological analyses. To measure the pulmonary medial wall thickness, sections were stained with Mayer’s Hematoxylin & Eosin. Stained sections were imaged using an Axio Imager Z1 upright microscopy system (Carl Zeiss, Oberkochen, Germany). Of the pulmonary arteries (< 120 µm in diameter), 20 were randomly selected in each animal (n = 3 per group), and the medial wall thickness was calculated as previously reported^47^: (medial thickness × 2)/external diameter × 100.

### Masson’s trichrome staining

For the assessment of perivascular fibrosis, sections were stained with Masson’s Trichrome ((MT, cat. HT15; Sigma-Aldrich, St. USA)). Perivascular fibrosis was determined by the area of the MT-stained fibrotic area divided by the short vessel diameter using ImageJ software (NIH, Bethesda, USA).

### 3,3-Diaminobenzidine (DAB) staining

Lung paraffin block tissues were sectioned at 7 μm, placed on slides, and dried at 37 °C for 24 h. Slides were the paraffin embedded, incubated in 0.3% H_2_O_2_ (cat. H1009; Sigma-Aldrich, St. USA) for 30 min, rinsed 3 times with PBS, incubated in animal serum to block antibody binding, incubated with primary antibodies (sTable 1), and then rinsed 3 times with PBS. They were then treated with biotinylated secondary antibodies in the ABC kit (cat. PK-6100; Vector Laboratories, USA), incubated for 1 h in blocking solution, and rinsed 3 times with PBS. Slides were developed with DAB (3,3-diaminobenzidinem, cat. D8001; Sigma-aldrich, USA) substrate for 12 min, mounted with cover slips and DPX mounting solution (cat. 06522; Sigma-Aldrich, USA), and visualized by light microscopy (Olympus Optical Co., Japan). The average number of PCNA-positive cells was counted in 5 small arteries that were randomly chosen (< 120 µm in diameter) using ImageJ software (NIH, Bethesda, USA). The percentage of PCNA-positive cells was calculated by dividing the number of PCNA-positive cells by the total cells^48^.

### RNA extraction and cDNA synthesis

The total RNA within cells and tissues was isolated using RNAiso Plus (cat. 9108, TAKARA, Japan) according to the manufacturer's instructions. 0.5 ml of RNAiso Plus was mixed with 0.1 ml of chloroform and incubated at room temperature for 7 min. Afterward, centrifugation is performed for 15 min at 4 °C at 12,000 × g. The supernatant is collected in a new tube, mixed with 0.25 ml of 100% isopropanol, gently shaken and then centrifuged again to precipitate the RNA. The supernatant was discarded and the submerged RNA pellet was washed with 70% ethanol and centrifuged at 7500×*g* for 5 min at 4 °C. The dried pellet was dissolved in 30 μl of diethyl pyrocarbonate (DEPC, cat. W2004; Biosesang, Korea) water and RNA was quantified using Nanodrop 2000 (Thermo Fisher Scientific). RNA was synthesized into cDNA using the cDNA synthesis kit (cat. 6210A; Takara, Japan)^48^.

### Quantitative real-time PCR

Total RNA and cDNA were prepared from rat lungs as described above. cDNA was used for quantitative real-time PCR (qRT-PCR) using SYBR green (cat. RR820A; Takara, Japan). All primers were purchased from Cosmogentech (Seoul, Korea). Relative mRNA levels of the genes were normalized to *β-actin *(*Atcb*), and the relative expression differences were obtained using the 2^-ddCt^ method^49^.

### Microarray experiments

Gene expression profiles were generated for lung tissues obtained from CON and MCT + Saline rats, as well as MCT + Saline rats injected with AD-, BM-, and UCB-MSCs using an Agilent-028279 Rat 8 × 60 k chip, which includes 62,976 probes corresponding to 18,584 annotated genes (Agilent, Santa Clara, CA, USA), according to the manufacturer’s instructions^50^. In each condition, we performed gene expression profiling of two biological replicates obtained from independent rats. Total RNA was extracted from rat lung using Trizol reagent (Invitrogen Life Technologies, Grand Island, NY). The integrity of the total RNA was analyzed using an Agilent 2100 Bioanalyzer. The RNA integrity values for all samples were larger than 8.8. Reverse transcription was then performed to generate cRNA, which was amplified and hybridized onto each array according to the manufacturer's instructions. The array was scanned using a SureScan Microarray Scanner (Agilent).

### Analysis of gene expression profiles

Probe intensities from the arrays were converted to log_2_-intensities and normalized using the quantile normalization method^51,52^. Differentially expressed genes (DEGs) were identified using an integrative statistical method as previously described^53,54^. Briefly, for each gene, we calculated a T-statistic value using a Student’s t-test and a log_2_-median-ratio test for each comparison. Empirical distributions of the T-statistic values and log_2_-median-ratios for the null hypothesis that the genes were not differentially expressed were estimated by performing random permutations of the samples and by then applying the Gaussian kernel density estimation method to the T-statistic values and log_2_-median-ratios that resulted from the random permutations. Using the empirical distributions, we computed the adjusted *P* values for the t-test and log_2_-median-ratio test for each gene and then combined these *P* values with Stouffer’s method^54^. False discovery rates (FDRs) of each gene for the combined *P* values were then computed with the Storey method^53^. Finally, the DEGs were defined as the genes with an FDR ≤ 0.05 and an absolute log_2_-median-ratio ≥ 0.58 (1.5-fold change).

### Heat map generation and GOBP enrichment analysis

The DEGs were categorized into 8 groups (G1–8) based on their up- or down-regulation patterns in the four comparisons: (1) MCT + Saline/CON, (2) MCT + AD/MCT + Saline, (3) MCT + BM/MCT + Saline, and (4) MCT + UCB/MCT + Saline. In each of the four major groups (G1–4), the DEGs were then sorted according to their up-regulation, no change, and down-regulation patterns in the order of the four comparisons. The heat map for the DEGs in G1–4 was finally generated using their log2-fold-changes in the four comparisons with “imagesc.m” in MATLAB (R2019a; www.mathworks.com/). Additionally, enrichment analysis of the gene ontology biological processes (GOBPs) for a set of the DEGs was performed using the DAVID software^21^. The GOBPs represented by the DEGs were defined as those with an enrichment *P* value < 0.05 calculated by DAVID, where *P* is the significance of the GOBPs being represented by the DEGs in G1–4. The enrichment *P* values were converted into − log10(P), and the heat map for the enrichment significance of GOBPs was generated using their − log10(P) values with “imagesc.m” in MATLAB (R2019a; www.mathworks.com/).

### Network analysis

To reconstruct a network model, we first selected a subset of the DEGs involved in the traditional pathways related to PAH progression (the endothelin pathway, NO pathway, and prostacyclin pathway). We then collected the protein–protein interactions (PPIs) among the selected DEGs from five protein–protein interactome databases: the biomolecular interaction network database^22,55^, human protein reference database^22^, biological general repository for interaction datasets^56^, molecular INTeraction database^57^, and search tool for recurring instances of neighboring genes^58^. To map human proteins onto the rat proteins, we used human-rat orthology information in mouse genome informatics^59^. The PPIs for the selected DEGs in the “sif” format were imported into Cytoscape (v.3.3.0; www.cytoscape.org/)^60^. The node attributes, including the log2-fold-changes in the four comparisons and gene symbols, in an attribute excel file were then imported into Cytoscape. Finally, the node and boundary color gradients were set using the continuous mapping of the log2-fold-changes. The nodes in the network model were arranged manually based on the localizations and relationships of the corresponding proteins in the Kyoto Encyclopedia of Genes and Genomes pathway database^61^. The network model was exported from Cytoscape in the PDF format. After opening the PDF file in Adobe Illustrator CS6 (version 16.0.0; www.adobe.com), additional edges identified from the KEGG pathway database and the previous literature were included to the network model.

### Statistical analysis

Non-parametric analysis was used given the small samples available. Comparisons were made using the Mann–Whitney U test. Significant differences are indicated as follows; by an asterisk (*) versus control, $ versus MCT + saline, and # versus MCT + UCB. Results are presented as means ± SDs and experiments were performed in triplicate. The analysis was conducted using SPSS version 22 (IBM Corporation, Armonk, NY).

## Supplementary information

Supplementary Information.
