# Supplementary material for: Comparative analysis on the anti-inflammatory/immune effect of mesenchymal stem cell therapy for the treatment of pulmonary arterial hypertension
Source: Sci Rep. 2021 Jan 21;11:2012. doi: 10.1038/s41598-021-81244-1 (PMC7820276; doi:10.1038/s41598-021-81244-1)
Supplement: Supplementary file 1 — Supplementary Information. [file 41598_2021_81244_MOESM1_ESM.docx]

**Comparative Analysis on the Anti-inflammatory/immune Effect of Mesenchymal Stem Cell Therapy for the Treatment of Pulmonary Arterial Hypertension**

Seyeon Oh^*^, Albert Y. Jang^*^, Sehyun Chae^*^, Seungbum Choi, Jeongsik Moon, Minsu Kim, Edda Spiekerkoetter, Roham T. Zamanian, Phillip C. Yang, Daehee Hwang^⸶^, Kyunghee Byun^⸶^, Wook-Jin Chung^⸶^

⸶ These authors contributed equally to this work as co-corresponding authors.

* These authors contributed equally to this work as co-first authors.

**Supplementary methods**

**Characterization of human mesenchymal stem cells**

AD-, BM-, and UCB-MSCs were all cultured in Dulbecco’s Modified Eagle’s Medium-low glucose media mixed with 10% mesenchymal stem cell growth supplements as previously described^1^. MSCs at passage 2 were characterized by flow cytometry using antibodies against human MSC markers, *CD44* and *CD90*. MSCs were also stained with an antibody against *CD34*, a negative marker for MSCs^1,2^. Antibodies were purchased from BD Pharmingen (BD Pharmingen, NJ, USA).

**Supplementary table 1. List of antibodies for immunostaining**

| Antibody name | Company | Cat. No. | Immunofluorescence |
| --- | --- | --- | --- |
| PCNA | Abcam | Ab2426 | 1:250 |
| CD80 | Santa-cruz | Sc-28347 | 1:100 |
| CD206 | Santa-cruz | Sc-58987 | 1:100 |
| TNF-α | Santa-cruz | Sc-52746 | 1:200 |
| TGF-β | abcam | Ab46715 | 1:250 |
| CD8 | Santa-cruz | Sc-1177 | 1:100 |
| CD20 | Santa-cruz | Sc-393894 | 1:100 |
| IL-8 | Santa-cruz | Sc-376750 | 1:100 |
| IL-10 | abcam | Ab189392 | 1:200 |

**Supplementary figure**


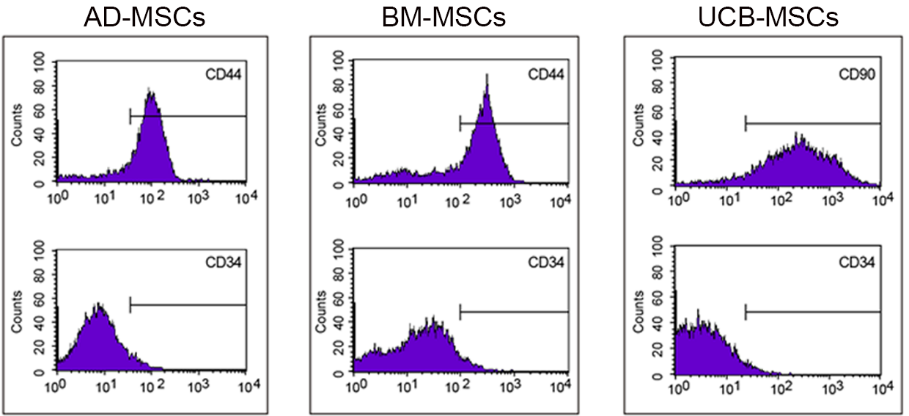


**sFig. 1 Characterization of the MSCs.** MSCs were characterized by flow cytometry using human MSC markers, *CD44* and *CD90* (top row), and a negative MSC marker, *CD34* (bottom row) (n=3 per group). AD-MSCs, adipose tissue derived mesenchymal stem cells; BM-MSCs, bone marrow derived mesenchymal stem cells; UCB-MSCs, umbilical cord blood derived mesenchymal stem cells


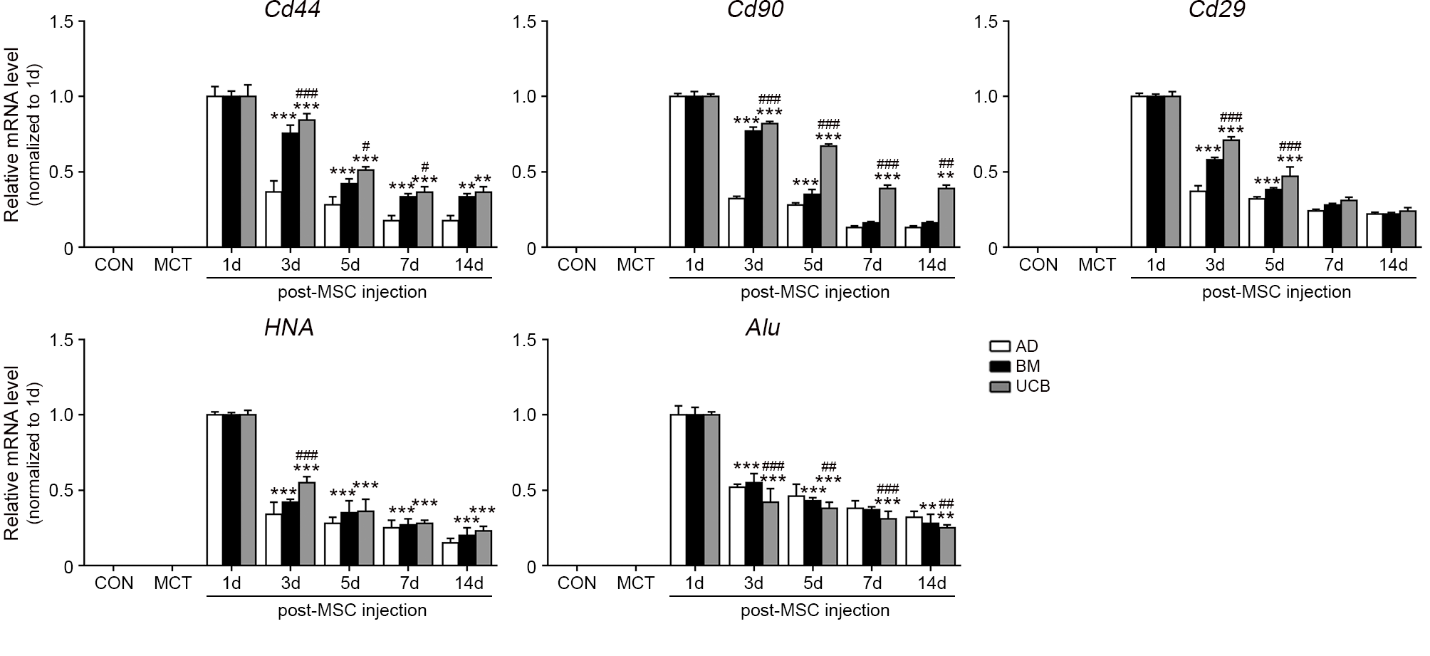


**sFig. 2 Survival of the engrafted MSCs in rat lungs. MSC engraftment in lung was assessed by measuring the mRNA levels of the human MSC markers *Cd44*, *Cd90*, *Cd29, HNA, and Alu* in lungs isolated at days 1, 3, 5, 7, and 14 post-injection**. The expression levels were normalized with respect to those in the control group. The normalized data are shown as the mean ± SD. * for comparison with CON; # for comparison with MCT+AD; *, # p < 0.05; **, ## p < 0.01; ***, ###, p < 0.001 by Mann-Whitney test. MSC, mesenchymal stem cells; MCT, monocrotaline; AD, adipose tissue derived; BM, bone marrow derived; UCB, umbilical cord blood derived, HNA, human nuclear antigen; Alu, Arthrobacter luteus.


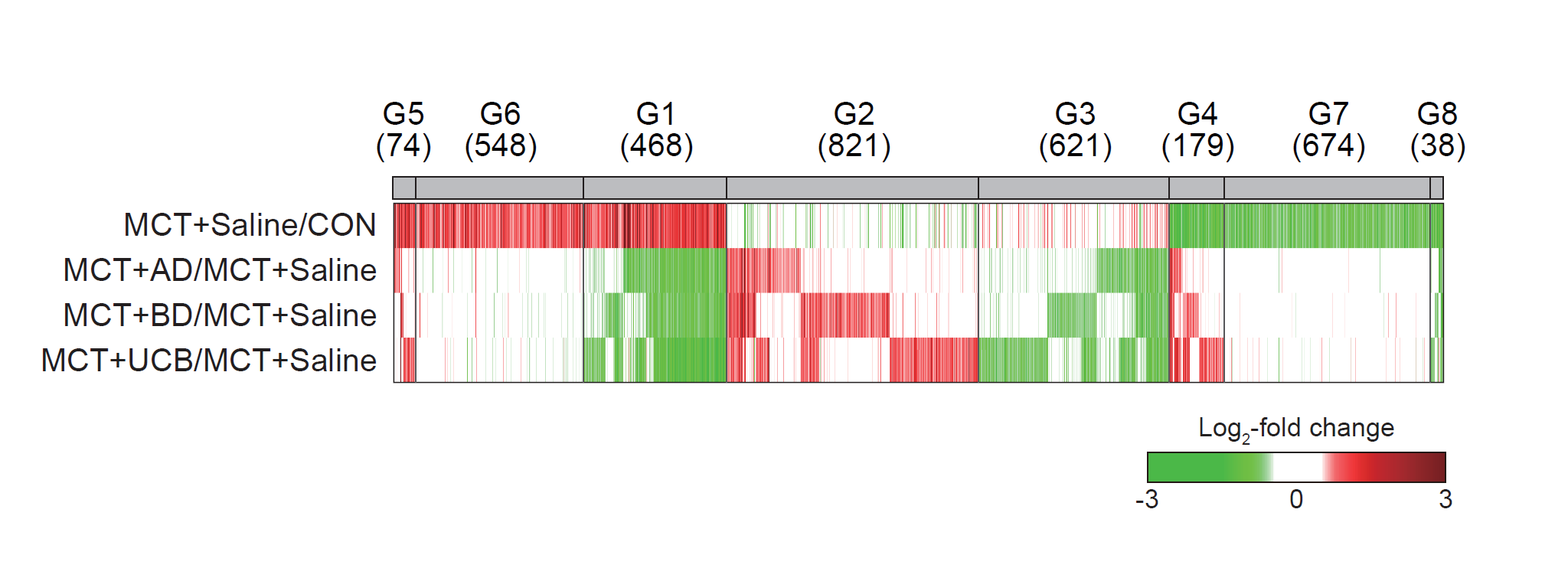
**sFig.3 Eight clusters of 3,423 DEGs identified by their differential expression patterns from the four comparisons (MCT+Saline/CON, MCT+AD/MCT+Saline, MCT+BM/MCT+Saline, and MCT+UCB/MCT+Saline).** Colors represent the increase (red) and decrease (green) of mRNA expression levels in the four comparisons. The number of genes in each cluster is shown. The heat map was generated using MATLAB (imagesc.m in R2019a; www.mathworks.com/). MCT, monocrotaline; CON, control; AD, adipose tissue derived; BM, bone marrow derived; UCB, umbilical cord blood derived


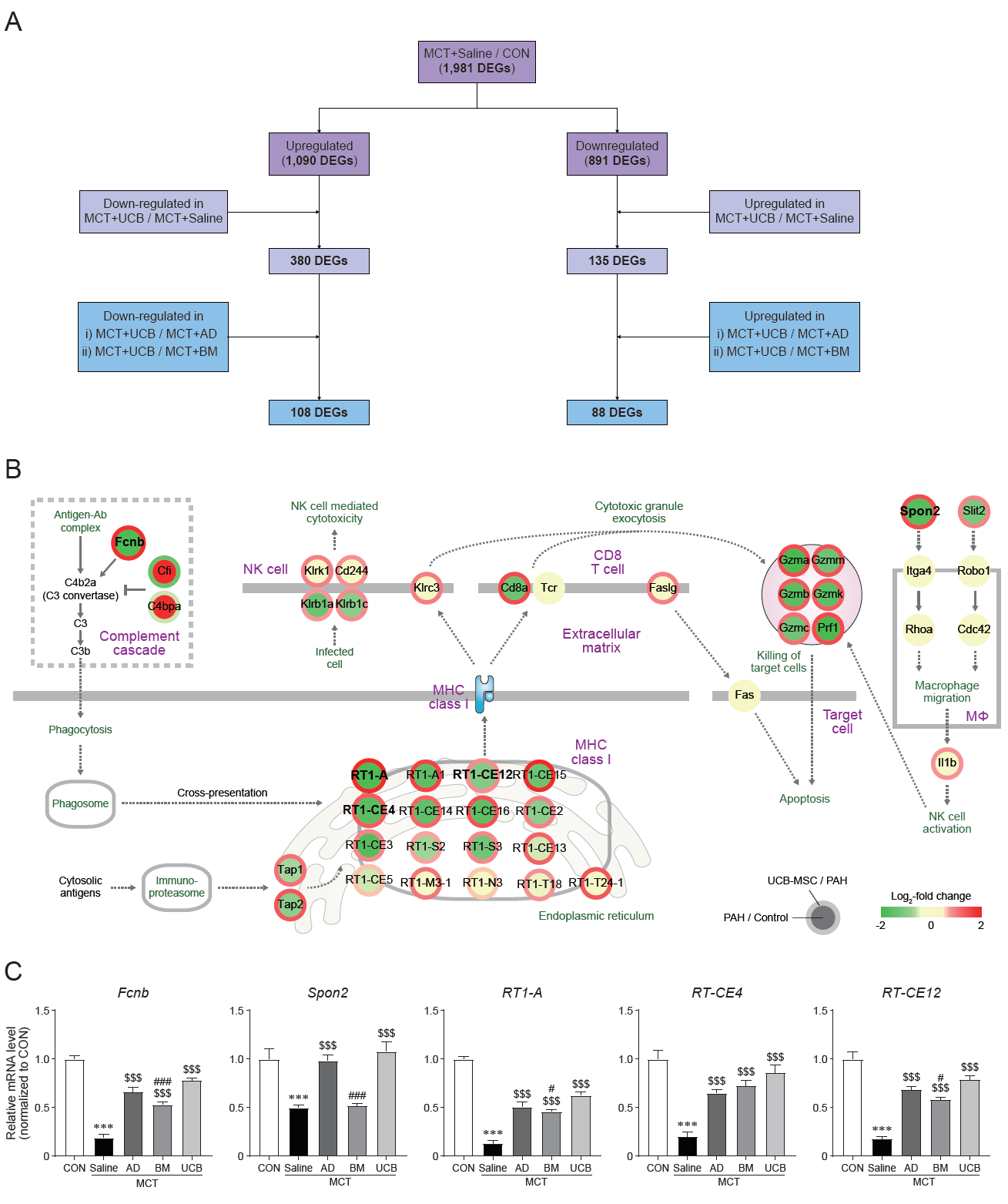
**sFig. 4 Enrichment analysis of the differentially expressed genes affected by the UCB-MSC treatment. a** The overall scheme for selection of 108 upregulated and 88 downregulated genes predominantly by UCB-MSCs, compared with by AD- and BM-MSCs. **b** A network model describing interactions among the predominantly downregulated genes by UCB-MSCs. Node center and boundary colors represent upregulation (red) and downregulation (green) by MCT and UCB-MSCs, respectively. Solid and dotted arrows (or inhibition symbols) denote direct and indirect activations (or inhibition), respectively. The color bar represents the gradient of log_2_-fold-changes in the corresponding comparison (MCT+Saline /CON or MCT+UCB/MCT+Saline). The network model was generated using Cytoscape software (v.3.3.0; [www.cytoscape.org/](http://www.cytoscape.org/)). **c** RT-PCR analysis confirming predominant downregulation of the representative genes involved in killing of target cells in the MCT+UCB group, compared with in the MCT+AD and BM groups. The expression levels were normalized with respect to those in the CON group. The normalized data are expressed as the mean ± SD. * for comparison with CON; $ for comparison with MCT+Saline; # for comparison with MCT+UCB; *, $, # P < 0.05 ; ***, $$$, ### P < 0.001 from Mann-Whitney test. MCT, monocrotaline; CON, control; AD, adipose tissue derived; BM, bone marrow derived; UCB, umbilical cord blood derived


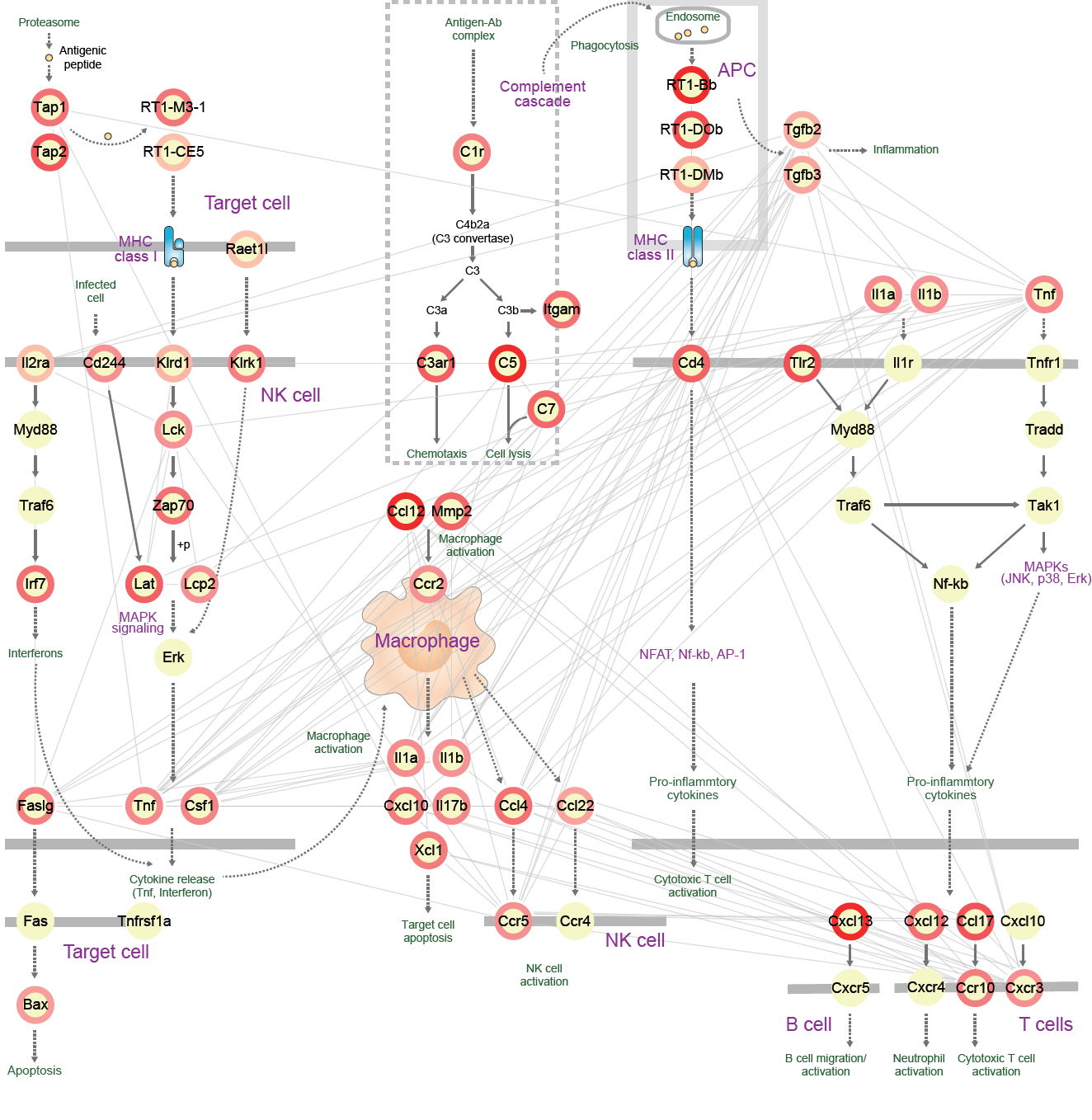
**sFig 5.** **A network model describing interactions among the genes not affected by MCT, but upregulated by MSCs.** Among the genes in G2 in Fig. 4b, the genes involved in immune and inflammatory responses were used for the network modeling. Node center and boundary colors represent upregulation (red) and downregulation (green) by MCT and MSCs (AD, BM, or UCB), respectively. Solid and dotted arrows (or inhibition symbols) denote direct and indirect activations (or inhibition), respectively. The color bar represents the gradient of log_2_-fold-changes in the corresponding comparison (MCT/CON, MCT+AD/MCT, MCT+BM/MCT, or MCT+UCB/MCT). The network model was generated using Cytoscape software (v.3.3.0; www.cytoscape.org/).

**Supplementary reference**

1. Kern S. *et al.* Comparative analysis of mesenchymal stem cells from bone marrow, umbilical cord blood, or adipose tissue. *Stem Cells*. **24**, 1294-1301 (2006).

2. Jin H.J. *et al.* Comparative analysis of human mesenchymal stem cells from bone marrow, adipose tissue, and umbilical cord blood as sources of cell therapy. *Int J Mol Sci.* **14**, 17986-18001 (2013).
